# RTAIAED: A Real-Time Ambulance in an Emergency Detector with a Pyramidal Part-Based Model Composed of MFCCs and YOLOv8

**DOI:** 10.3390/s24072321

**Published:** 2024-04-05

**Authors:** Alessandro Mecocci, Claudio Grassi

**Affiliations:** Department of Information Engineering and Mathematical Sciences, University of Siena, 53100 Siena, Italy; claudio.grassi@student.unisi.it

**Keywords:** ambulance detection, smart traffic light, YOLOv8, emergency detection, siren detection, MFCCs, real-time detector

## Abstract

In emergency situations, every second counts for an ambulance navigating through traffic. Efficient use of traffic light systems can play a crucial role in minimizing response time. This paper introduces a novel automated Real-Time Ambulance in an Emergency Detector (RTAIAED). The proposed system uses special Lookout Stations (LSs) suitably positioned at a certain distance from each involved traffic light (TL), to obtain timely and safe transitions to green lights as the Ambulance in an Emergency (AIAE) approaches. The foundation of the proposed system is built on the simultaneous processing of video and audio data. The video analysis is inspired by the Part-Based Model theory integrating tailored video detectors that leverage a custom YOLOv8 model for enhanced precision. Concurrently the audio analysis component employs a neural network designed to analyze Mel Frequency Cepstral Coefficients (MFCCs) providing an accurate classification of auditory information. This dual-faceted approach facilitates a cohesive and synergistic analysis of sensory inputs. It incorporates a logic-based component to integrate and interpret the detections from each sensory channel, thereby ensuring the precise identification of an AIAE as it approaches a traffic light. Extensive experiments confirm the robustness of the approach and its reliable application in real-world scenarios thanks to its predictions in real time (reaching an fps of 11.8 on a Jetson Nano and a response time up to 0.25 s), showcasing the ability to detect AIAEs even in challenging conditions, such as noisy environments, nighttime, or adverse weather conditions, provided a suitable-quality camera is appropriately positioned. The RTAIAED is particularly effective on one-way roads, addressing the challenge of regulating the sequence of traffic light signals so as to ensure a green signal to the AIAE when arriving in front of the TL, despite the presence of the “double red” periods in which the one-way traffic is cleared of vehicles coming from one direction before allowing those coming from the other side. Also, it is suitable for managing temporary situations, like in the case of roadworks.

## 1. Introduction

**Figure 1:**
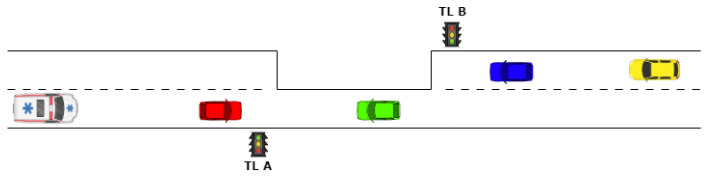
Unidirectional traffic managed by a traffic light system.

In the domain of emergency response, the efficiency of emergency vehicles is paramount to ensure timely assistance to individuals in life-threatening situations. Rapid deployment and arrival of these vehicles, such as ambulances, fire trucks, and police cars, can significantly influence the outcome of emergencies. Among the numerous challenges these vehicles face, one of the most critical is navigating through traffic swiftly. Studies by Chowdhury et al. [1], Xie et al. [2], and Vishal et al. [3] have identified key factors contributing to response delays, including traffic congestion, geographical barriers, and operational inefficiencies. Specifically, ambulances encounter unique challenges in their mission to provide immediate medical care. The urgency of reaching patients or transporting them to healthcare facilities necessitates overcoming any delay that could adversely affect patient outcomes. Recognizing traffic congestion as a primary hurdle, particularly at traffic signals and one-way restraints, our research introduces a viable solution aimed at mitigating these delays. Our proposed system is designed to significantly reduce, if not entirely eliminate, delays encountered by Ambulances in an Emergency (AIAEs) at traffic lights, in particular in one-way street restrictions or roadwork. Contributions by Rangel et al. [4] highlight the critical impact of traffic light delays on ambulance response times, underscoring the need for effective solutions. By enabling a rapid green light sequence for ambulances, our system aims at enhancing the overall efficiency of emergency medical services, thereby improving patient survival rates and response effectiveness in urban and sub-urban settings. In the absence of policies that promptly grant ambulances a green light, critical risks emerge. In fact, ambulance drivers, when confronted with a red traffic signal during emergencies, face a dire choice: delay by waiting for the green light, potentially jeopardizing patient outcomes, or proceed with caution through the red light, increasing the risk of vehicular accidents. This dilemma is underscored by data indicating a significant number of accidents annually at traffic lights due to emergency vehicles disregarding red signals, highlighted in both media reports and academic studies [5,6,7]. Research by Ray and Kupas [8] further substantiates the heightened risk of ambulance-involved accidents at intersections, noting these incidents often result in greater harm and involve more individuals compared to similar accidents with non-emergency vehicles. Kahn et al. [9] also reveal a stark correlation between emergency operations and the incidence of crashes and fatalities, underscoring the urgent need for a solution. The implementation of an intelligent system capable of granting timely green lights to ambulances could mitigate such risks, enhancing the safety and efficiency of emergency medical responses. Additionally, an adaptive traffic management system benefits regular traffic flow, reducing the broader impacts of traffic congestion, including excessive fuel consumption, time wastage, and increased carbon emissions [10]. Traditional fixed-time traffic signals contribute significantly to these inefficiencies, a challenge that can be addressed by adopting intelligent traffic management strategies, as demonstrated by Mousavi et al. [11], Zhao et al. [12], and others. While the bulk of existing literature on traffic management focuses on traditional four-way intersections, our research extends this knowledge base by exploring the management of unidirectional traffic flows—often a requirement due to roadworks or various restrictions, as illustrated in Figure 1. Unidirectional flows are characterized by extended periods of red lights under fixed-time control policies, a necessary measure to ensure safe crossings when green lights are eventually activated. These extended red light phases, which do not typically apply to intersection settings, are critical for the effective management of unidirectional traffic. Our paper addresses the unique challenges posed by these traffic conditions, proposing an intelligent traffic management system specifically designed to minimize response times and improve safety for ambulances navigating these complex environments.

**Figure 2:**
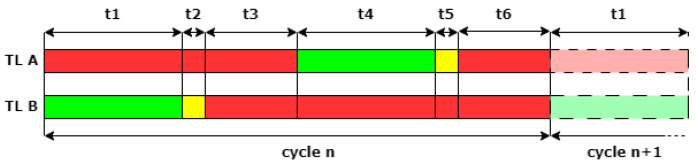
Traffic light cycle to manage unidirectional traffic, using standard color indications: green for proceed, yellow as a caution to stop if it is safe, and red to halt under all conditions.

Figure 2 illustrates a schema of the traffic light (TL) cycle in the case of unidirectional traffic management, showing the operation of traffic lights using standard color indications: green for proceed, yellow as a caution to stop if it is safe, and red to halt under all conditions.

During interval t1, only vehicles from Traffic Light B (TLB) are allowed to enter and cross the restricted one-way area. Following this, during t2, these vehicles are signaled with a yellow light, indicating caution, while Traffic Light A (TLA) displays a red light to prevent entry into the one-way area. The interval t3, termed as the “double red” period, is essential for ensuring the one-way area is cleared of vehicles moving from TLB towards TLA, prohibiting new entries during this time. Conversely, interval t4 permits vehicles from TLA to enter and cross the one-way area, followed by a cautionary yellow phase in t5, with TLB showing red to restrict access. The “double red” period in t6 ensures vehicles within the one-way area can safely reach the other side, specifically towards TLB, while preventing new entries. The critical “double red” periods, t3 and t6, ensure the safe, alternate, one-directional flow of traffic within the restricted area. These intervals account for the time necessary for vehicles to complete the crossing at regulated speeds, ensuring the area is cleared before the traffic flow changes its direction. The duration of these “double red” intervals is dictated by the length of the restricted area and the enforced speed limit, crucial for maintaining vehicular safety, and hence, is fixed and non-negotiable.

Our solution focuses on modifying intervals t1 and t4 to accommodate Ambulances in an Emergency (AIAEs), while maintaining the minimum legally required durations for t2 and t5, as discussed in Section 3.2. This ensures that vehicles from TLA completing their crossing during these phases can do so safely, without affecting the overall safety of road usage within this segment. Such adaptive measures can significantly decrease the likelihood of prolonged waiting times for ambulances, particularly in situations where they approach an intersection at the tail end of the yellow signal phase for their travel direction.

Our proposed Real-Time Ambulance in an Emergency Detector (RTAIAED) utilizes a combination of elementary detectors within an architecture inspired by the “part-based model” framework, facilitating swift and accurate detections. This novel system not only facilitates the timely activation of green lights for ambulances on approach, but also presents a scalable solution that improves the efficiency of emergency responses without the need for supplementary infrastructure.

The RTAIAED system integrates video and audio analysis within a unified framework, utilizing a feedforward neural network for audio processing and multiple deep learning detectors based on YOLOv8 for video analysis. This combination enhances the system’s performance through a synergistic effect, where a logic-based segment plays a pivotal role in orchestrating the outputs from the deep learning models. It processes and refines these outputs, facilitating a translation into insights that are readily understandable by human operators within the context of the RTAIAED’s application. To the best of our knowledge, this parallel and synergistic approach to audio and video analysis represents an innovative contribution to the AIAE scenarios.

The main contributions of this paper are outlined as follows:

1. The development of a new architecture (called RTAIAED), inspired by the Deformable Part Model [13], capable of accurately and reliably detecting Ambulances in Emergency. The system is composed of some elementary detectors based on deep learning, operating in synergy, each specialized in the recognition of a specific sub-part. It can be seen as a revamped and simplified version of the Deformable Part Model with a binary penalty function (with a value 0 if the part is detected inside the root, i.e., the ambulance body, or 1 if the part is detected outside the ambulance body). This simplified positional description has been experimentally found effective because a more precise localization of the parts provides no additional relevant information. Moreover, unlike previous systems that operate by using video or audio separately, the RTAIAED fuses audio and video information to reach accurate and reliable detection. The system runs in real time even on an average single-board computer.
2. The development of a Lookout Station (LS) capable of running the RTAIAED at a certain distance from the corresponding traffic light. The early detection of an AIAE can be communicated remotely to grant the timely action of the traffic light controller (TLC) to minimize (or even cut to zero) the waiting time of the ambulance as it reaches the one-way restricted road.
3. A methodology to change the traffic light cycle in the unidirectional traffic case, so as to reducing the ambulance waiting time while preserving the safety of the involved vehicles in transit in the restricted tract.

The rest of this paper is organized as follows. Section 2 provides a review of related works to contextualize our approach within a broader scope, examining those works that address similar issues but with different methods. Section 3 presents the proposed methodology, the software, and the hardware involved. Section 4 reports the experimental results and Section 5 reports the conclusions and future work.

## 2. Related Works

In the current literature, the focus of optimization of the traffic light systems is put on the case of intersection management. To enhance the behavior of traffic lights in comparison to the fixed-time policy, the contemporary literature frequently turns to reinforcement learning techniques, as exemplified by prior work of Liang et al. [14] or Wiering et al. [15].

Some works, like that of Liu et al. [16], employ reinforcement learning with multi-agents to orchestrate all the traffic lights in a city (or at least those at adjacent road intersections), achieving significant results in terms of decongestion. However, it is noteworthy that these approaches do not prioritize emergency vehicles. Also, the orchestration between multiple traffic lights is not applicable to the unidirectional traffic flow management, given that they are usually in isolated areas far from the other traffic light systems and a lot of them are only temporary (e.g., in the case of roadworks), so not directly employable in a solution that considers every traffic light system of a city.

Other approaches, like that of Navarro-Espinoza et al. [17], attempt to predict future traffic flow (the next 5 min) based on past flow (the last hour). Nonetheless, they neglect to exploit the current traffic flow to acquire information about the prospective flow in an alternative location. The foresight into the anticipated arrival time of each detected vehicle yields noteworthy advantages, particularly in scenarios where, at a specific moment, a substantial number of vehicles are observed arriving from one side while none or significantly fewer are coming from the other.

Another important aspect is linked to the communication between the traffic light controller and the “outside world”. To notify the traffic light controller of an approaching AIAE, it must be equipped with a receiver so that the controller itself can adjust the sequence and duration of the lights upon receiving specific signals and commands. These signals may be transmitted either by a human operator or by an automated system utilizing various sources (radio, audio, visual) to identify the presence of an AIAE. In the “VAI 118” system [18], for example, constant communication takes place between the Urgency and Medical Emergency Service (SUEM) operations center and the Municipality’s Mobility Center. Ambulance arrivals are signaled using on-board Personal Digital Assistants (PDAs), requiring each ambulance to be consistently equipped with a functional PDA. Ford has also proposed a comparable solution [19], which is currently in progress, wherein the emergency vehicle communicates directly with the traffic light upon its approach. However, this approach alerts the traffic light when the emergency vehicle is already in close proximity. Ford’s strategy involves reducing the need for a complete stop by decreasing the vehicle’s speed through proprietary software. This ensures that by the time the vehicle reaches the traffic light, the signal will have already switched to green. This does not solve the problem at its root, given that the waiting time at the traffic light is shifted to a longer travel time to reach it. Both of these solutions necessitate the installation of a dedicated device in every ambulance, which would entail additional hardware for both traffic lights and ambulances. As a result, scaling these solutions could pose significant challenges. Siddiqi et al. [20] proposed a system in which the communication between ambulances and traffic lights is provided through a mobile application developed by them and operated by ambulances teams. This application controls the traffic lights via SMS. However, all these solutions require the remote manipulation of traffic lights by human operators. Moreover, neither of these approaches address the objective of minimizing traffic congestion. In contrast, Islam et al. propose an alternative approach [21] in which only the traffic lights require additional hardware to detect emergency vehicles. Their solution entails installing multiple microphones to detect the presence of a siren and determine its direction. This approach eliminates the need for additional equipment in each ambulance and addresses some limitations of the previously mentioned solutions. A more accurate approach relying solely on audio data has been introduced by Pacheco-Gonzalez et al. [22]. However, these approaches do not guarantee the reliable detection of an ambulance’s arrival at a traffic light, as the siren sound may propagate from parallel roads, and adverse weather conditions and ambient noises can obscure the ambulance’s presence. Additionally, these methods cannot be leveraged to alleviate traffic congestion, since they are not able to obtain information about the current traffic flow. In an alternative strategy, using visual data, Barbosa et al. [23] developed a system to distinguish between different types of vehicles (ambulances, police cars, fire trucks, buses, and regular cars), assigning each a different priority. However, their system does not verify if the emergency vehicles are genuinely in an emergency, prioritizing them even when unnecessary. Also, their solution does not integrate the visual data with audio data. Nonetheless, their algorithm is considered to be put at the traffic light, resulting in the inefficient minimization of the waiting time of an ambulance in the case of a “double red” period of a one-way road.

In conclusion, another alternative approach uses Radio Frequency Identification (RFID), a technique that uniquely identifies objects by exploiting radio waves. This method was proposed by Sharma et al. [24], in which the emergency vehicles broadcast messages to signal their presence, allowing the messages to be read by the traffic light controller (TLC). This approach requires the installation of a radio frequency reader on each road managed by a traffic light system and an RFID tag on each emergency vehicle, resulting in a not-so-well-scalable model. Similarly, in the solution by Eltayeb et al. [25], each ambulance and TLC is equipped with a Global System for Mobile Communications (GSM) module (with a SIM card) to communicate via SMS the position of the ambulance, thanks to a GPS embedded in the vehicle.

Existing approaches predominantly center around vehicles currently positioned at the traffic light. What distinguishes RTAIAED is fourfold. Firstly, it does not require any additional hardware in the ambulances; secondly, it emphasizes the estimation of the expected arrival time of vehicles identified at a considerable distance, enabling prompt and proactive actions; thirdly, it is very reliable in detecting a genuine AIAE (Ambulance in an Emergency), discarding the ambulances not in an emergency and the ones not going towards the traffic lights; fourthly, it can be easily adapted to also manage the traffic flow in normal conditions without additional hardware.

Regarding the architecture of RTAIAED, it draws inspiration from the “Parts and Structure” concept inherent in Part-Based Models (PMs), first introduced by Fischler and Elschlager in 1973 [26]. These models detect the presence of an object of interest in an image by analyzing various parts within the image. An evolution of Part-Based Models is the Constellation Model, proposed by Fergus et al. [27], wherein a small number of features, their relative positions, and mutual geometric constraints are leveraged to determine the presence of an object of interest.

Utilizing subpart discovery, the RTAIAED enhances the robustness of its detection capabilities. Our approach simplifies the mutual positioning constraints by implementing binary functions that maintain essential data, thereby reducing computational load. Deep learning models [28] serve as the primary detectors within this framework, distinguishing relevant parts and representing a departure from conventional Part-Based Model (PM) stochastic learning approaches. Moreover, the RTAIAED integrates audio analysis with video and temporal data through a layered methodology, culminating in a comprehensive decision-making process. This multidimensional approach ensures a more accurate and computationally efficient detection process.

## 3. The RTAIAED: HW and SW Architecture

In laying the groundwork for the implementation of the proposed RTAIAED, it is crucial to take into account three key factors: the passage of an ambulance, the existence of an emergency situation, and the direction of travel. To improve the system performance, we consider the fact that a typical ambulance incorporates distinctive elements, like:

- the symbol of the star of life;
- the symbol of the red cross;
- the written text “ambulanza” (mirrored or not).

**Figure 3:**
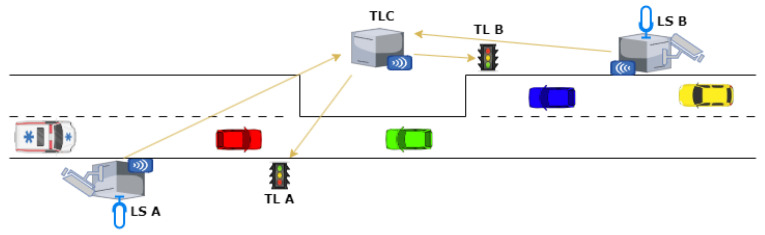
Unidirectional traffic managed by a traffic light system with the proposed solution, with the arrows showing the direction of communication between devices.

Even if the previous elements relate to Italian ambulances, similar distinctive elements are present in all ambulances around the world and the general approach proposed in this paper can be easily adapted by training the recognition modules to detect and identify each specific set of elements. Additionally, the direction of travel is pivotal; only ambulances approaching the traffic light should receive a green signal. Moreover, in emergency scenarios, ambulances will activate their flashing lights and sirens. All these elements, except the siren, can be captured through images. Processing the siren signal, however, necessitates audio capabilities. Hence, a camera and a microphone are essential components. Furthermore, it is imperative to obtain this information from a distance, specifically at an LS, necessitating the inclusion of a transceiver module. Another crucial constraint is obtaining real-time information. As we will show, a single-board computer is sufficient for this objective. Referring back to the preceding statements, the RTAIAED is built upon these essential software and hardware components and it is expected to be mounted at each LS, as shown in Figure 3. In it, each LS communicates the presence of an AIAE to the traffic light controller (TLC), to enable the manipulation of the sequence of colors and their duration at the traffic lights at both ends of the one-way restriction. The camera of each LS is deliberately pointed in the opposite direction of the road restriction, to be able to catch the AIAE as far as possible from the tract of unidirectional traffic (i.e., as early as possible).

### 3.1. SW Architecture

To accurately detect AIAE, the presence of an ambulance with its peculiar elements, its motion direction, and the status of its siren must be assessed. The RTAIAED is thus based on several elementary detectors, which can be divided into two fundamental modules:

- some visual object detectors, which process in real time the frames coming from the camera and are based on some customized YOLOv8 models, each of them with the task of detecting a component of the ambulance or the ambulance in its entirety, distinguishing its front part from its rear part;
- an audio detector, which processes in real time the audio coming from the microphone, exploiting a feedforward neural network based on Mel Frequency Cepstral Coefficients (MFCCs).

**Figure 4:**
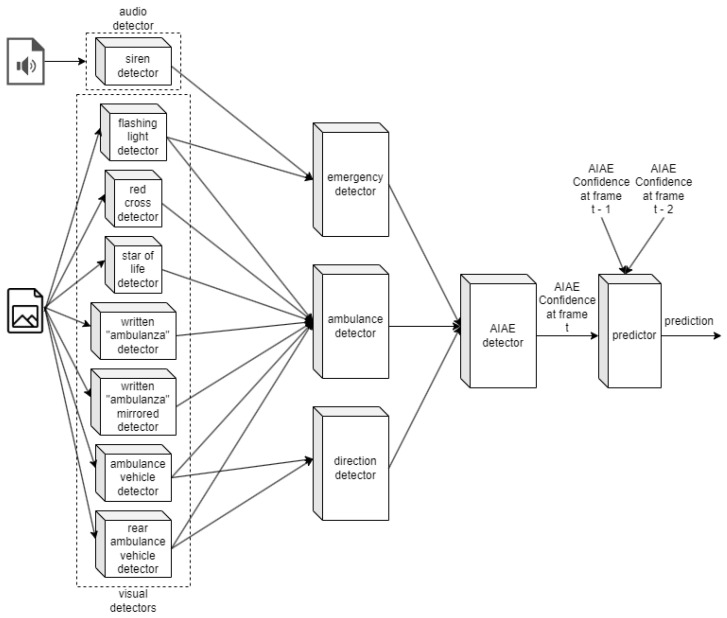
Architecture of the RTAIAED.

In the RTAIAED system, each elementary detector operates simultaneously but independently. For every video frame analyzed, the visual object detectors provide immediate predictions specific to that frame. Concurrently, the audio detector processes signals every 1.5 s, with their findings remaining applicable to all video frames within this interval until the next audio analysis cycle begins. The system’s architecture, illustrated in Figure 4, adopts a hierarchical design that integrates these elementary audio and visual detectors. These detectors contribute their findings towards the decisions made by three specialized detectors—dedicated to identifying emergencies, ambulances, and their direction of travel. The cumulative data from these detectors inform the Ambulance in an Emergency (AIAE) detector, which calculates a confidence score indicating the likelihood of an AIAE presence in the video frame being analyzed. This decision-making process involves synthesizing the results from the current and the preceding two video frames. In essence, the system confirms the presence of an AIAE based on the consistent detection of an ambulance, moving in the appropriate direction towards the traffic lights, across at least three consecutive frames. This confirmation is further contingent on the simultaneous identification of emergency indicators, such as sirens or flashing lights, that underscore the emergency’s urgency.

In the following, each part composing the model is discussed in this order: the elementary audio detector, the elementary visual detectors, the ambulance detector, the emergency detector, the direction detector, the AIAE detector.

#### 3.1.1. The Elementary Audio Detector

The audio detector, also referred to as a siren detector, is employed to detect the typical sound of an ambulance siren, with the primary objective of being a pillar for the emergency detector. It gives its prediction based on a input time-frame comprising the last two seconds of the audio collected by the microphone and it is executed every 1.5 s, providing a partial overlap of 25% between two consecutive input time-frames to catch, in a timely manner, sirens starting at the end of a 2 s sample. In particular, it has been developed by extracting Mel Frequency Cepstral Coefficient (MFCC) features from two-second audio samples and feeding them into a feedforward neural network. MFCCs have been chosen due to their ability to offer a condensed representation of the spectrum of an audio signal, encapsulating information about rate changes across various spectrum bands [29]. Other studies employing the MFCCs for the siren sound detection show that two-second samples are long enough to give high accuracy for the detection [30].

The NN used to detect the siren has the following architecture:

- four fully connected layers;
- three dropout layers, each of which randomly sets input units to 0 with a predetermined frequency to prevent overfitting;
- three reLu;
- a final softmax, to decide on the two output classes (siren or nothing).

**Figure 5:**
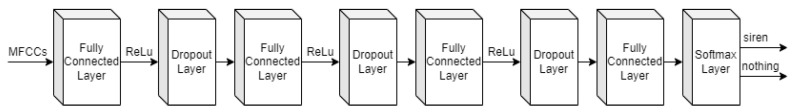
Architecture of the siren detector.

In Figure 5, a schema of the NN is reported.

The siren detector has been implemented by using Tensorflow and Keras and then it has been exported to the ONNX format. The training dataset comprises 1240 .wav files featuring the presence of a siren (one or more), alongside 4805 files with silence or different kinds of sounds (airplanes, trains, rain, storms, chainsaws, helicopters, traffic noises, anti-theft alarms, fireworks, animal sounds). For training purposes, 90% of the dataset has been used, while the remaining 10% has been reserved for validation.

#### 3.1.2. The Elementary Visual Detectors

The elementary visual detectors play a crucial role in accurately detecting the ambulance vehicle and its parts within video frames. Additionally, they help disambiguate the direction of the ambulance, enhancing overall detection precision. The parts to be detected are:

- the ambulance itself, where the front and the rear of the ambulance are detected as separate parts;
- the flashing light;
- the symbols of the star of life and of the red cross;
- the text “ambulanza”, in the normal or reversed sense.

The front part and the rear part of the ambulance have two different labels to distinguish when an ambulance is approaching the traffic light and when it is going away from it. These detectors have been created with a customized YOLOv8 model. YOLO is a deep learning model introduced in 2015 by Redmon et al. [31]. It is a cutting-edge deep learning model designed for real-time object detection. It rapidly identifies specific objects in videos, live feeds, or images. Leveraging deep convolutional neural networks, YOLO extracts features from input images, enabling the prediction of bounding boxes with confidence scores for each recognized object. Its speed is a result of segmenting an image into smaller units, processed only once by the algorithm: an innovation compared to multi-stage or region-based methods. YOLO models, including the latest iteration, YOLOv8, developed by Ultralytics [32], can be efficiently trained on a single GPU, ensuring high accuracy while retaining a compact size.

Under YOLOv8, there are multiple models of different sizes, ranging from smaller to larger, namely YOLOv8n, YOLOv8s, YOLOv8m, YOLOv8l, YOLOv8x. All YOLOv8 models come pre-trained on the COCO dataset [33], a comprehensive collection of images spanning 80 different object categories. By default, the size of the input images is set at 640 × 640 pixels, but any size multiple of 32 is accepted. For the elementary visual detectors, the model n has been exploited as a starting point, leading to a customized model with less parameters, similar to that demonstrated by Ke et al. [34] in the domain of license plate recognition or by Xiao et al. [35] in the domain of pedestrian detection and tracking. The customized model (the tiny model t) has been created by reducing the number of channels and layers in the final part of the backbone, passing from 225 layers, 3,012,213 parameters, 3,012,197 gradients, 8.2 GFLOPs of the model n (occupying 5.93 MB) to 218 layers, 1,357,013 parameters, 1,356,997 gradients, 6.3 GFLOPs (occupying 2.76 MB).

Both model n and model t have been trained with two different input resolutions: 640 × 640 pixels and 480 × 480 pixels, leading to the creation of the models 640n, 640t, 480n, 480t. For simplicity, only the results of the best model in terms of speed and final performance will be reported (i.e., 480t), while providing aggregated comparisons with the other models. Each candidate to be the elementary visual detector (i.e., the models 640n, 640t, 480n, 480t) has been trained on a dataset containing 2172 images, collected from photos and videos with heterogeneous resolutions, including day and night scenes, different weather conditions, and various urban and rural environments to ensure the robustness of our system. This diversity aims to closely reflect the expected real-world operating environment of the system. Every object has been manually labelled with the tool label Img [36], drawing a rectangle around each of them, taking inspiration from the work by Xiong et al. [37].

In addition to labeled images, the dataset includes background images devoid of labeled objects, strategically incorporated to mitigate false positives. The model 640n underwent iterative training using incremental learning, with evaluations conducted after each iteration to pinpoint instances of suboptimal performance. Following each iteration, new images resembling those where the model exhibited shortcomings were introduced, enhancing the model’s proficiency in addressing these specific scenarios. Using the model 640n as a starting point, further iterations have been incorporated to refine and train the models 640t and 480n. Thereafter, the insights gained from model 480n have been leveraged to develop the derivative model 480t.

**Figure 6:**
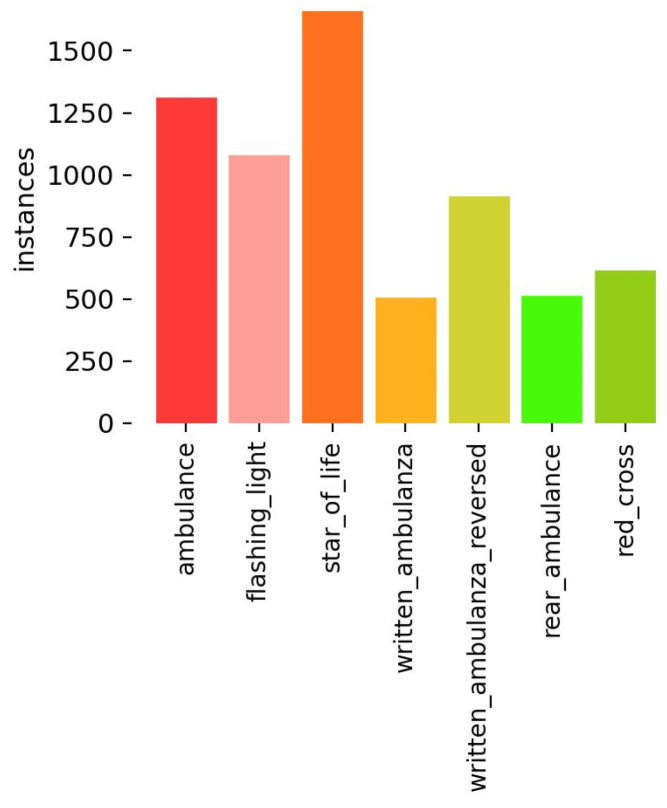
Histogram of the instances used in the training dataset.

The distribution of the instances used in the training dataset is shown in the histogram of Figure 6.

#### 3.1.3. The Ambulance Detector

**Figure 7:**
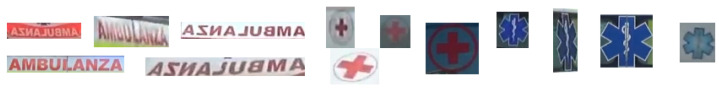
Written texts and symbols shown as they appear in the frames.

The so-called ambulance detector takes as input the results from all elementary visual detectors and undergoes additional processing to confirm the presence of at least one ambulance within the scanned frame. More specifically, it checks whether the elementary ambulance vehicle detector has predicted the presence of an ambulance. In the affirmative scenario, the remaining elementary visual detectors are considered; otherwise, no further action is taken, and no ambulance presence is assumed. If the additional detectors identify some specific sub-parts, they adjust the confidence that an ambulance is truly present within the current frame based on their findings. The positions of all predicted sub-parts are scrutinized for location consistency. Specifically, the flashing light detection concentrates on the upper portion of a recognized ambulance. Instances where flashing lights are not detected in this region are filtered out and labeled as false positives. Regarding written or symbolic elements (such as “ambulanza” and “ambulanza” reversed, where “ambulanza” is the Italian term for “ambulance”, the symbol of the star of life, and the symbol of the red cross, illustrated in Figure 7), they are considered correctly detected only if they appear within a region recognized as an ambulance vehicle. Detections outside this context are filtered out. Additionally, only for the standalone testing of this detector does the detection of the rear part of an ambulance result in the prediction being discarded, while it is employed in a more complex way in the Direction Detector.

Each detected element that survives the filtering process is then assigned a weight, which is subsequently multiplied by its corresponding confidence score from the object detector’s prediction. The formula used to determine the presence of an ambulance is as follows:

(1) $$f\left(c\right)=\sum_{i=1}^{N}w_{j}c_{i}\left(j\right)>T_{amb}$$

In this formula, $T_{amb}$ signifies the confidence threshold necessary to classify a prediction as an ambulance, considering all detected components. Nrepresents the total number of detected elements associated with an ambulance. The term $c_{i}\left(j\right)$ refers to the confidence level of the prediction for the ith element within class j, sourced from deep visual models. Each model, representing an elementary visual detector, correlates to a unique class. The variable $w_{j}$ denotes the weight assigned to the jth class, indicating the relative importance of that class in the detection process. The presence of multiple instances of the same class within a single image, such as several stars of life, aids in surpassing the $T_{amb}$ threshold. This method, inspired by Deformable Parts Models [13], is critical in ensuring the identified ambulance corresponds with the expected characteristics of such a vehicle, suggesting that a higher number of ambulance-specific elements within a detection area increases the probability of accurate identification.

The threshold and weights can be determined empirically or through alternative methods, such as employing a dynamic programming algorithm to assess each final prediction generated by the model based on various thresholds and weights. Eventually, the best-performing combination is selected according to a predefined criterion.

The trade-off arises from the fact that reducing the threshold increases the likelihood of detecting more true positives but also leads to an uptick in false positives. Therefore, the threshold should not be set excessively low (to avoid an excess of false positives) nor too high (to prevent overlooking genuine ambulances).

In our solution, three thresholds (0.8, 0.85, and 0.9) have been considered, and the weights have been learned according to the following formula:

(2) $$Min\sum_{t=1}^{T}\left(FPr_{t}-TPr_{t}^{2}\right)$$

The objective of this formula is to find the weights that minimize the FPr (False Positive rate of the presence of an ambulance going towards the camera) while maximizing the TPr (True Positive rate) squared found for each of the T thresholds (0.8, 0.85, and 0.9). TPr is squared to assign it less significance in comparison to FPr, given that both values range between 0 and 1. Squaring TPr reduces its impact, considering the possibility that an unnoticed ambulance can be identified in subsequent frames. The emphasis lies in avoiding the error of predicting an object as an ambulance when it is not, rather than missing the recognition of an actual ambulance. Going into detail, Formula (2) can be rewritten in the following way:

(3) $$Min\sum_{t=1}^{T}\left(FPr_{t}-TPr_{t}^{2}\right)=Min\left(\sum_{t=1}^{T}\sum_{s=1}^{S}\sum_{i=1}^{N}w_{j}{\bar{c}}_{s_{i_{t}}}\left(j\right)-w_{j}^{2}c_{s_{i_{t}}}^{2}\left(j\right)\right)$$

In it, S is the number of samples (images) on which the weights are learned, N is the number of objects detected in each sample, and j is the class of the detected object. $\bar{c}$ denotes the confidence of an object inside an image without an ambulance going towards the camera that has produced a false positive, along with the other objects inside the same image using Formula (1), according to the threshold t, and c denotes the confidence of an object in an image containing one or more ambulances going towards the camera that has produced a true positive according to the threshold t. The weights w follow the constraints:

(4) $$w_{j}\in \left[-1.0,1.0\right]$$

(5) $$w_{j}=0.05\cdot z$$

with z ∈Z.

The weights have been learned using a dataset with challenging samples of ambulances or vehicles that may resemble ambulances. The dataset comprises 559 images with hard-to-recognize ambulances, 501 images without them, and 320 images of the rear part of an ambulance. The output of this detector is the overall confidence of the presence of an ambulance, according to Formula (1).

#### 3.1.4. The Emergency Detector

Even when an ambulance is visible in the scene, it is crucial to reliably assess whether it is in an emergency state or not (i.e., if the ambulance has its flashing lights and siren on). The assessment of an actual emergency is carried out every video frame by exploiting the results coming from the siren detector and the flashing light detector. Concerning the flashing lights, due to their intermittent nature, even if they are on, they do not emit light continuously and they can be detected by the flashing light detector only in those video frames where they emit light, so it can happen that they are detectable in one frame but not in the next one. To mitigate this fact, the emergency detector, upon a detection of the flashing light detector, retains this information for the subsequent 4 frames. It is important to retain and propagate this information to the following frames (where the flashing light may not emit light even if in a state of emergency) because to obtain the confirmation of an AIAE, at least three consecutive video frames must predict the combined presence of ambulance, emergency, and correct direction of motion. Concerning the siren, since the siren detector is executed every one-and-a-half seconds, therefore having a slower sampling rate than visual detectors, its result is retained by the emergency detector for all video frames until the next execution of the siren detector itself. Therefore, the emergency detector combines the outcomes of the flashing light detector and the siren detector to raise a flag indicating the presence of an emergency. This information is crucial for determining whether an Ambulance in an Emergency (AIAE) is approaching a traffic light. The output of this process includes a flag indicating the predicted emergency and also conveys information about the presence or absence of the siren.

#### 3.1.5. The Direction Detector

As mentioned in Section 3.1, the direction in which an Ambulance in an Emergency (AIAE) is moving holds vital information for traffic management, specifically in deciding if its passage should be expedited. However, AIAEs moving away from intersections are not prioritized. To ascertain the ambulance’s direction, the system looks for signs of the ambulance moving closer, such as not showing its rear to the camera and its size increasing in the video frames; therefore, it undergoes two checks:

- Intersection over Union (IoU) Test: This test compares detections from two detectors: one for the ambulance’s front (from the ambulance vehicle detector) and another for its rear (from the rear ambulance detector). If both detectors identify the same vehicle, the detection is discarded as incorrect, typically for ambulances that are far away.
- Size Comparison: By comparing the ambulance’s size across frames, the system can determine if it is moving towards the traffic light (and thus the camera), expecting ambulances to appear larger as they approach due to the camera’s perspective.

The output is a simple flag indicating the predicted direction. The accuracy of the predicted direction is crucial, as it determines the necessity for immediate traffic light adjustment. Specifically, only ambulances approaching the traffic light warrant an expedited transition to green, in contrast to those moving away. Therefore, it is vital to effectively exclude any AIAE that is receding from the camera’s viewpoint, which, given the camera’s strategic installation at the Lookout Station (LS), correlates with moving away from the traffic light. This differentiation ensures that the system’s response is appropriately targeted and efficient, facilitating rapid passage for ambulances heading towards emergencies while minimizing unnecessary disruptions to the normal flow of traffic.

#### 3.1.6. The AIAE Detector

This final detector, i.e., the Ambulance in an Emergency Detector (AIAE detector), takes the outputs of the emergency detector, ambulance detector, and direction detector, to formulate the ultimate prediction. Specifically, the emergency detector and the direction detector must have their respective flags raised, indicating the detection of an emergency and the correct motion of the ambulance towards the traffic light. If these conditions are met, the overall confidence output by the ambulance detector is harnessed and stored in memory, becoming the output of the Ambulance in an Emergency (AIAE) detector.

Only if, for three consecutive frames, the confidence returned by the AIAE detector exceeds the threshold $T_{amb}$ (introduced in Formula (1)) is the presence of an AIAE stated. There are cases where it is likely that an ambulance is actually present. In these cases, some small bonuses help in reaching the threshold (acting as a lowering of the threshold). The bonuses in these cases are:

- Siren Detection Bonus: If a siren is detected by the emergency detector, a slight boost of 0.05 is added to the confidence score. This is based on the rationale that the audible presence of a siren increases the likelihood of an ambulance being in the scene.
- Successive Frame Bonus: Sometimes it can happen that partial occlusions or other interfering phenomena actually reduce the confidence of the detected ambulance in the subsequent frames (even if it has been initially detected), so the three consecutive detections to confirm the final presence could not be reached. In order to stabilize this behavior, the bonus is applied after the first detection of the ambulance so that it can be more easily detected in the subsequent frames. Specifically, this bonus is equal to the confidence of the ambulance detected in the previous frame multiplied by 0.008, and it is added iteratively to the additional bonus of the previous frame. This means that for every frame in which the ambulance remains visible, this additional bonus aids the system in recognizing the ambulance and, only if the other conditions are also satisfied (correct direction and spotted emergency), predicting an AIAE.

The correct values of these bonuses have been tested and chosen empirically.

In the tests and experiments with the Real-Time Ambulance in an Emergency Detection (RTAIAED), the threshold $T_{amb}$ used is 0.85. This choice has been made considering the results of the standalone test for the Ambulance Detector in Section 4.3, from which it emerges that $T_{amb}$ at 0.90 is too restrictive, losing too many True Positives of ambulances, while at 0.80, too many False Positives occur. Further extensive tests on the overall RTAIAED have shown that $T_{amb}$ at 0.85 with the use of the bonuses is both accurate and quick in giving the final decision.

#### 3.1.7. SW and HW Implementation of the LS

The whole set of previously described detectors has been developed using Python 3.8 (and its libraries) on a laptop running Windows 10 Home, with an NVIDIA RTX 3080 GPU, an AMD Ryzen 9 5900HX with Radeon Graphics 180 3.30 GHz CPU, and 16 GB of RAM. Thereafter, the LS has been implemented and tested both on a Jetson Nano, equipped with NVIDIA Maxwell 182 GPU, Quad-core ARM Cortex-A57 Processor, and 4GB LPDDR4 Memory, and on a Raspberry Pi 5, featuring a 64-bit quad-core Cortex-A76 Processor with 8GB LPDDR4X Memory. All this equipment has been enclosed in a robust IP68 box, and connected to a camera (Arducam IMX519) and a microphone (MillSO USBPCMIC558), thus forming an instance of the Lookout Station (LS).

### 3.2. Optimal Distance of the Lookout Stations

To manage a one-way traffic scenario (for example, in a rural environment where a certain road has been restricted due to repair works), we assume the presence of two traffic lights controlling alternate vehicle access. These traffic lights are positioned at both ends of the restricted tract, as depicted in Figure 1. In this setup, two Lookout Stations (LSs) are suitably positioned nearby the controlled area. One station is positioned at a certain distance before one of the two traffic lights and the other is positioned before the second traffic light on the other side, as illustrated in Figure 3. Their primary objective is to reliably detect every AIAE approaching the restricted track from either side, thanks to the RTAIAED mounted on them. This information is then transmitted to the traffic light controller, to adjust the timing of the two traffic lights accordingly.

Upon detecting an AIAE at a strategically placed LS, referring to the time cycle of Figure 2, our system adjusts t1 or t4 to ensure the ambulance encounters a green light upon arrival. Specifically, if an AIAE approaches from TLA towards TLB, as depicted in Figure 3, the system acts dynamically as follows:

- If the AIAE is detected at the LS during t1 (which usually is significantly bigger than the “double red” periods), t1 itself is reduced to zero and t2 (the yellow duration) is set to the minimum required by law.
- If the AIAE is detected at the LS during t2, t2 itself is set to the minimum required by law.
- If the AIAE is detected at the LS during t3, no action is taken.
- If the AIAE is detected at the LS during t4, t4 itself is extended by a certain amount to ensure that when the AIAE arrives in front of TLA, the green light is still present.
- If the AIAE is detected at the LS during t5, the cycle is rewound, returning to t4, whose duration is extended to ensure that when the AIAE arrives in front of TLA, the green light is still present.
- If the AIAE is detected at the LS during t6, the cycle is rewound, returning to t4, whose duration is extended to ensure that when the AIAE arrives in front of TLA, the green light is still present.

Independently of when the Ambulance in an Emergency (AIAE) is identified by the Lookout Station (LS), the system ensures it arrives at Traffic Light A (TLA) during the green phase of t4, allowing for immediate passage through the restricted area. This optimization leverages the travel time between the LS and the traffic light to guarantee that the non-negotiable t3 phase has concluded (applicable when the AIAE is detected during t1, t2, or t3). In scenarios where detection occurs outside these intervals, the system sufficiently extends or reverts to t4 to maintain green light availability. Importantly, the adjustments made to t5 and t6 simply shift these intervals within the cycle without shortening their duration. Furthermore, this adaptive approach applies symmetrically when an ambulance approaches from the opposite direction, ensuring a green light at the traffic light regardless of the direction of approach. It turns out that the worst case is when there is a green light on the other side (t1 for ambulances spotted at the LSA) because of the unsuppressible double red time before giving the green light to the right side, and it is the reason for the need to find an optimal position for the Lookout Stations.

The optimal position of the Lookout Stations depends on the speed of the ambulance (e.g., 90 km/h in the majority of instances), the minimum duration time of yellow light allowed by the regulations (e.g., in Italy, according to “risoluzione del Ministero dei Trasporti n. 67906 del 16.7.2007” [38], 3, 4, and 5 s, respectively, for the speed limits of 50, 60, and 70 km/h on the road), and the duration time of the double red. This last parameter is established by companies specializing in the design, supply, and installation of traffic light systems. Their goal is to deliver a secure product customized for the particular road where the system is deployed. However, for our discussion and to provide a broad understanding of the distances involved, it can be simplified as contingent on the separation between the two traffic lights placed at the ends of the constriction section. Additionally, it is influenced by the speed of the vehicles passing through the one-way road, and estimated according to the following formula:

(6) $$DoubleRedTime=\frac{RoadLength}{VehicleSpeed}$$

The required time for safely changing the traffic light under the worst-case scenario where the green light is on the opposite side of the road in relation to the AIAE (ensuring the segment between the two traffic lights is entirely clear of cars) is as follows:

(7) RequiredTime=DoubleRedTime+YellowTime

The calculated required time stems from the consideration that, in the worst-case scenario, a yellow light is immediately activated on the opposite side, followed by the red light. Once the red signal is initiated on the other side, the double red time commences, and only upon its completion can the green light be activated on the side of the AIAE.

Some examples of the optimal Lookout Station position with respect to the nearest traffic light (optimal distance) are shown in Table 1.

The optimal distance is based on the assumption that the ambulance is traveling at a speed of 90 km/h, as per the following formula:

(8) OptimalDistance=RequiredTime·AmbulanceSpeed

This way, putting the Lookout Station at the optimal distance, it is guaranteed that in the worst case, according to the hypothesis, the ambulance will arrive at the traffic light when the green light is firing at it, given that the time required for the traffic light to switch the lights safely is the one required for the ambulance at high speed to reach the nearest traffic light from the Lookout Station.

Another issue that arises pertains to how much time the green light should be maintained after the detection of an AIAE. This is crucial, as the green signal must persist at least until the ambulance has reached and moved beyond the location of the nearest traffic light to the LS. Two possible approaches can be used for achieving this: installing another station at the traffic light to monitor the ambulance’s position as it passes, or, without adding any extra station, estimating a sufficiently extended time to ensure the ambulance has reached and moved past the traffic light. The minimum duration time of the green light depends on the two following scenarios:

1. MinGreenTime1, if there is already a green light towards the AIAE, calculated with the formula:
  (9) $$MinGreenTime1=\frac{OptimalDistance}{AmbulanceLowSpeed}$$
2. MinGreenTime2, if there is a red light towards the AIAE that has to be switched to a green light according to the required time of Formula (7):
  (10) $$MinGreenTime2=\frac{OptimalDistance}{AmbulanceLowSpeed}-RequiredTime$$

Table 2 shows these minimum times for the duration of the green light taking the examples shown in Table 1 and assuming the ambulance’s low speed is 40 km/h; this ensures its passage even in cases where it cannot travel at high speeds.

### 3.3. Communication with the Traffic Light Controller

Considering that the distances between the Lookout Station (LS) equipped with the RTAIAED and the nearest traffic light can exceed 200 m, a long-range wireless solution is necessary to transmit the alarm signals. To optimize the implementation costs, the LoRa technology [39] has been selected for its cost-effective modules and its ability to offer a low-power solution, reaching distances of up to 15 km with high immunity to interference. More specifically, LoRa transceiver modules, such as the SX1276 from Semtech, have been designated for placement at the traffic light controller (operating as a receiver) and at each LS (operating as a transmitter).

### 3.4. Evaluation Metrics

The elementary detectors are evaluated according to the following metrics, where TP stands for true positives, TN for true negatives, FP for false positives, and FN for false negatives. In particular, the accuracy has been used for the audio detector, calculated as follows:

(11) $$Accuracy=\frac{TP+TN}{TP+TN+FP+FN}$$

For the visual detectors, the metrics used are the precision and the recall, calculated as follows:

(12) $$Precision=\frac{TP}{TP+FP}$$

(13) $$Recall=\frac{TP}{TP+FN}$$

Also, the mAP50 and the mAP50-95 have been used. These metrics use the intersection over union (IoU), calculated as:

(14) $$IoU=\frac{AreaofIntersection}{AreaofUnion}\;$$

Basically, it is the ratio of the area of intersection between the predicted and ground truth bounding boxes to the area of their union. mAP is then calculated as:

(15) $$mAP=\frac{1}{N}\sum_{i=1}^{N}AP_{i}$$

In it, AP (standing for Average Precision) is the area under the Precision–Recall curve, and N is the number of object classes. mAP50 is obtained with mAP calculated for an IoU threshold of 0.5, while mAP50-95 is the mAP calculated over different IoU thresholds, from 0.5 to 0.95 with step 0.05.

## 4. Results

Every single part composing the RTAIAED has been tested in a standalone way. The overall results of the RTAIAED are then reported in Section 4.4, showcasing great performance despite the flaws of each detector taken individually.

### 4.1. Results of the Elementary Audio Detector

The following paragraph presents the results of the audio detector. In the validation phase, an accuracy of 99.5% was achieved after 150 epochs. During the testing phase, a distinct dataset was employed, encompassing 459 audio samples with sirens, 3368 audio samples containing various other sounds, and 52 synthetic audio samples. The synthetic audio samples were generated using Audacity 3.3.3 [40], combining siren sounds with other noisy and intense sounds. The results of the testing phase are as follows: 418 out of 459 sirens correctly detected (8.9% False Negatives), 3364 out of 3367 audio without sirens correctly detected (0.09% False Positives), 56 out of 65 sirens correctly detected in the synthetic audio samples (13.8% False Negatives).

Examining audio instances where sirens are not detected reveals scenarios characterized by excessively strong background noise or faint siren signals. Such cases are not deemed critical errors for the performance of the RTAIAED; the emergency is typically heard several seconds before the appearance of the ambulance in the camera, so, since sound is assessed every second and a half, instances where a siren is not detected at a specific moment are likely to be captured in the subsequent one. Moreover, even in the extreme case in which the siren remains undetected throughout an ambulance’s transit, the flashing lights can still be detected, ensuring an emergency detection in any case. False negatives do not pose a significant issue, as their occurrence without concurrent ambulance detection in the video does not lead to an AIAE detection.

### 4.2. Results of the Elementary Visual Detectors

The results of the model 480t on the evaluation dataset, containing 421 images, after the final iteration, are reported in Table 3, according to the metrics usually used with YOLO models: precision, recall, mAP50, and mAP50-95.

The data show the high performance of the ambulance’s class detection compared to the other classes. Also, the low values obtained for certain classes (in particular, the mAP50-95 of flashing light, written ambulanza, and red cross) are in line with or better than the values obtained in the recent literature with YOLOv8 in similar detection applications [41,42,43,44] (note that the official documentation of YOLOv8 [32] reports a 37.3% mAP50-95 on COCO with the model n).

Table 4 presents the consolidated results for all classes, sequentially listing the performance of models 640n, 640t, 480n, and 480t.

The performance of the 480t model is slightly inferior to that of the 640n model, with a small gap passing from the models with 640 pixels to the ones with 480 pixels. Nevertheless, owing to the implementation of the part-based pyramidal model within the comprehensive system, it manages to yield equivalent results in terms of final predictions compared to the other three models, as demonstrated later.

### 4.3. Results of the Ambulance Detector

The learned weights for each of the classes as input to the elementary visual detectors according to Formula (1) are as follows: 0.9 for the ambulance vehicle (denoting only the one moving toward the camera), −0.8 for the rear ambulance vehicle (indicating the one moving away from the camera), 0.5 for the flashing light, and 0.25 for the other elements. Using these weights and setting the threshold at 0.85, with the model 480t, only 12 images out of 501 without an ambulance (2.4%) resulted in false positives (from now on, denoted as FPO). Conversely, 405 images out of 559 featuring an ambulance were correctly identified as true positives (72.5%) (from now on denoted as TPA), while 7 out of 320 images featuring a rear ambulance were incorrectly predicted as ambulances (2.2%) (from now on, denoted as FPR).

Applying a threshold of 0.9 yielded the following outcomes. The FPO decreased to 10 from 12, marking a reduction to 2.0%, TPA dropped to 397 from 412, reflecting a significant decrease to 71.0%, and the FPR remained stable at 7, showing no change at 2.2%. Conversely, setting the threshold to 0.8 resulted in FPOs increasing to 14, up to 2.8%, TPAs rising to 427, indicating a substantial increase to 76.4%, and the FPR again remained unchanged at 7, equivalent to 2.2%. This pattern demonstrates that the incidence of false positives exhibits minimal variation, altering by less than 1% across different threshold settings, while the true positive count shows greater fluctuation.

In comparative analysis with other models, similar trends were observed, as detailed in Table 5, highlighting the outcomes across various thresholds. Given these findings, a threshold of 0.9 is deemed excessively stringent, leading to a notable loss of TPAs. Consequently, a threshold of 0.85 has been selected for implementation, which may be further adjusted downward, benefiting from enhancements introduced in Section 3.1.6.

**Figure 8:**
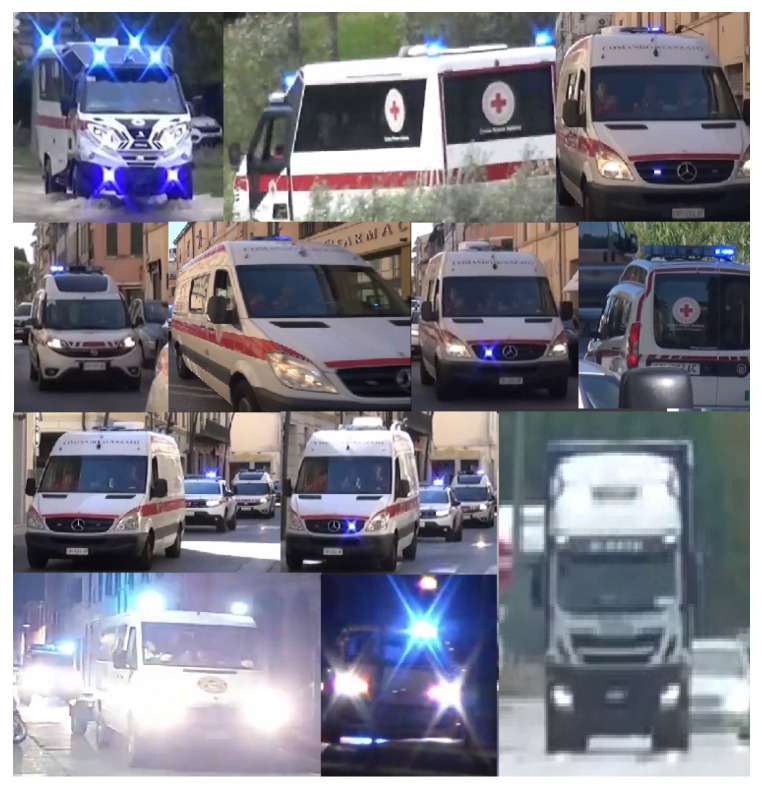
False positives: other vehicles identified as ambulances in at least one model.

Figure 8 reports the images comprising vehicles erroneously detected as ambulances. The data indicate that most cases involve vehicles that closely mimic the appearance and features of ambulances.

**Figure 9:**
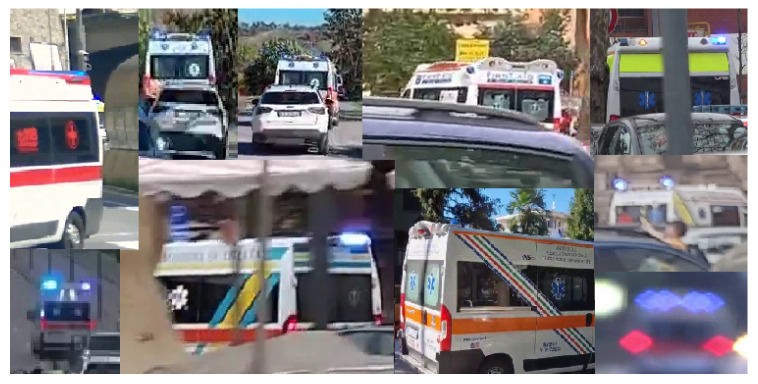
False positives: rear ambulances identified as ambulances (so, front part) in at least one model.

Figure 9 reports the images where the rear part of the ambulance is erroneously detected as its frontal part. Analysis reveals that the majority of cases are characterized by either central obstructions or the ambulance being significantly distant. So, in both cases, the majority of the remaining errors can be considered acceptable for the Ambulance Detector taken as a standalone detector, and they are the reason that all the components of the RTAIAED must work synergically to remove them.

**Figure 10:**
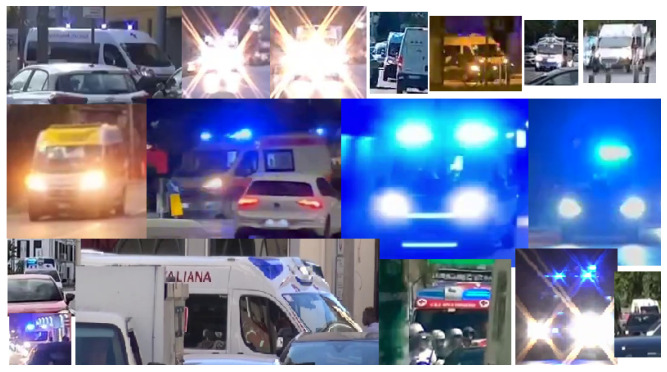
Some of the false negatives: ambulances not detected.

Also, the low rate of true positives (around 70–80% depending on the threshold and on the model) must not be considered as a problem because a lot of images were chosen containing ambulances really distant from the camera, with poor image quality, bad lighting effect, or too many obstacles in front of it, as shown in Figure 10. Even the occurrence of false positives in images devoid of an ambulance is not problematic. This is due to the fact that even if an object is mistakenly identified as an ambulance, it will be filtered out because neither flashing lights are detected nor a siren is heard, so the “false” ambulance is not regarded as an Ambulance in an Emergency (AIAE).

### 4.4. Results of the RTAIED

The entire system underwent testing on 85 videos, totaling 2312 s (i.e., almost 39 min), captured during both day (70) and night (15 videos). These videos contain 94 instances of Ambulances in an Emergency (AIAEs) along with other vehicles, including emergency vehicles with sirens and trucks resembling ambulances. Thirteen of these videos exclusively featured vehicles similar to ambulances, all equipped with flashing lights and/or sirens, ranging from fire trucks and police cars to blood transport and civil protection vehicles. Also, five ambulances not in an emergency are present. Each model of the object detector was evaluated on both the Jetson Nano and Raspberry Pi 5, using both default (the PyTorch one, with extension .pt, that is the official one) and ONNX formats. The smallest model, 480t, demonstrates comparable performance to other models, achieving the same detections due to additional checks implemented for consecutive frames. The RTAIAED using the 480t model produced zero false positives across all 85 videos, successfully detecting all 94 AIAEs approaching the traffic light. Notably, the system avoided misclassifying ambulances moving away from the camera as AIAEs approaching it, thanks to the double-check on the direction. Moreover, we successfully identified partially occluded AIAEs. Notably, AIAEs that were lined up and partly concealed by other vehicles were detected by focusing solely on the upper part of the vehicles. The siren of every AIAE with an activated siren was consistently recognized, typically preceding the AIAE’s appearance in the video. Although other emergency vehicles with activated sirens were detected, they were not classified as ambulances, resulting in the absence of AIAE detection. AIAEs equipped only with flashing lights were effectively identified, thanks to the detection of the emergency condition signaled by these lights. Remarkably, the RTAIAED demonstrated exceptional proficiency in detecting flashing lights during the night, facilitating the identification of a vehicle as an ambulance even when confidence was low due to limited lighting. This underscores the critical synergy between each component of the proposed RTAIAED.

To assess the standalone performance of the elementary ambulance detector and the elementary siren detector in predicting an AIAE, a specific test has been carried out. For the elementary ambulance detector, confidence thresholds of 0.85 and 0.90 have been used. In Table 6, the results of each individual detector are compared with those achieved by the overall RTAIAED, emphasizing the improvement of the RTAIAED in comparison to its elementary components.

The improvement becomes evident through the elimination of False Positives and False Negatives when employing the RTAIAED, in contrast to the occurrences observed with each elementary detector used in isolation. Specifically, when each elementary visual detector is utilized independently, it tends to misclassify all (5) non-emergency ambulances present in the videos as AIAEs. Additionally, adjusting the threshold for the elementary visual detector to classify something as an AIAE leads to a reduction in False Positives; however, this adjustment results in the omission of some AIAEs, causing an increase in False Negatives. In the case of the elementary siren detector, False Negatives were observed in instances where AIAEs had only the flashing light activated. Notably, False Positives were only registered when there was no AIAE present in the video, as the detector, when operating independently, fails to recognize the direction of ambulances. This results in a higher count of False Positives every time an AIAE moves in the opposite direction of the camera. Significantly, when examining each elementary detector in isolation, they exhibit comparable performance to models previously proposed in analogous studies [21,22,23] within their respective domains (audio or visual). The true advancement given by our system stems from the fusion of orthogonal predictions from these detectors. Each detector assesses distinct aspects compared to others, while also considering spatial relationships, such as the positioning of flashing lights atop an ambulance or the identification of characteristic elements within a silhouette recognized as an ambulance. The consolidation of the detection occurs only after three consecutive positive predictions, effectively mitigating false positives. Notably, upon testing the removal of the three-consecutive-frame rule introduced in Section 3.1.6, our system identified only two false positives among the 85 assessed videos: one involving a fire truck and the other concerning a civil protection vehicle. The incorporation of the three-consecutive-frame rule is practical primarily because our system operates in real time, capable of sampling multiple frames per second.

To demonstrate this, all YOLO models were also evaluated in terms of speed, and Table 7 presents the mean frames per second (fps) for each model using the Jetson Nano and Raspberry Pi 5. The model 480t exhibited the best results, both with the default model on the Jetson Nano and the ONNX model on the Raspberry Pi 5.

The model 480t is the preferred choice due to its high speed without compromising the accuracy of Ambulance in an Emergency (AIAE) detection. It consistently delivers satisfactory results; especially noteworthy is its capability to perform well on the Raspberry Pi 5. Despite the Jetson Nano achieving a 44% higher frames per second (fps) compared to the Raspberry Pi 5, the latter’s fps of 8.2 is still adequate for the RTAIAED. Furthermore, tests have shown that the response time of the RTAIAED employed in a Jetson Nano is less than 0.4 s in good-visibility conditions with spikes to 0.25 s, starting from the appearance of the ambulance in the video.

Tests further indicate that on a clear road without traffic, when an AIAE is moving at high speed, the detection by RTAIAED occurs 3-4 s before the AIAE disappears from the camera’s field of view, surpassing it. This highlights the RTAIAED’s ability to detect AIAEs even at a distance, providing crucial additional time to take timely proactive actions.

## 5. Discussion

The executed tests demonstrate the robust performance of the Real-Time Ambulance in an Emergency Detection (RTAIAED) system across diverse conditions. The pyramidal part-based system plays a crucial role in efficiently mitigating false positives generated by individual elementary detectors, highlighting the synergy among its multiple components in accurately detecting targeted objects while filtering out irrelevant information.

Our system operates under the assumption that the ambulance will proceed along the LS traffic light route without encountering significant delays, obstructions, or deviations onto alternate secondary roads. However, it is essential to acknowledge that these limitations mirror those of other systems, which may also be affected by additional constraints, such as restricted detection capabilities limited to the vicinity of traffic lights, thus not ensuring a timely transition to a green signal. Despite these considerations, our system maintains safety protocols in place. In instances where the LS encounters such scenarios, the primary consequence is the treatment of the ambulance as a conventional vehicle, resulting in adherence to traffic light signals like any other vehicle. Nevertheless, the likelihood of encountering these situations, particularly along LS traffic light sections spanning only a few hundred meters, remains relatively low, though not entirely absent. To address scenarios involving multiple roads leading to a traffic light located beyond the optimal distance for prompt signal adjustments, a separate LS should be positioned along each route to ensure timely responses.

Our solution has been implemented successfully for Italian ambulances, demonstrating its capability to detect all types currently in circulation in Italy. The adaptable nature of the concept allows for its potential extension to ambulances from other nations or states, incorporating specific elements or even expanding its application to other emergency vehicles by training the elementary object detector models accordingly.

Moreover, the architecture of the RTAIAED is versatile and can be applied to various domains where different features of an object of interest can be independently detected and then merged in a pyramidal manner to yield a robust final decision. While future developments and fine-tuning, such as speeding up with quantization or pruning techniques [45], are feasible, the current state establishes it as an operational product.

It is crucial to highlight the importance of optimal camera placement for achieving the best results. The camera should be positioned at a sufficient height to detect ambulances even when partially obstructed by vehicles or obstacles, facing the opposite direction compared to the traffic flow. Without the need for additional hardware, a simple modification to the object detector introduces two new classes for detecting vehicles in different directions of travel, paving the way for implementing solutions to alleviate traffic congestion.

The RTAIAED not only serves as an effective tool for emergency vehicle detection but also holds potential applications in intelligent traffic management. It can send signals to smart or autonomous vehicles in the proximity where an AIAE has been detected, alerting them or even lowering automatic road bollards when an AIAE is approaching.

### 5.1. Future Directions for Research and Development

The advancement of the Real-Time Ambulance in an Emergency Detection (RTAIAED) is poised to encompass several key initiatives:

- API Development for Enhanced Interoperability: Initiating the creation of a robust Application Programming Interface (API) will facilitate seamless data exchange between the RTAIAED and other intelligent systems. This will enable a broader spectrum of smart applications to leverage real-time information from the RTAIAED, enriching their functionality and responsiveness to emergencies.
- System Extension to Additional Emergency Vehicles: Inspired by the pioneering work of Barbosa et al. [23], efforts will be directed towards expanding the RTAIAED’s capabilities to incorporate a wider range of emergency vehicles. This enhancement aims to establish a systematic priority framework for various emergency services, optimizing their operational efficacy in critical situations.
- Exploratory Management of Conventional Traffic Flows: Currently in a conceptual phase, the potential for RTAIAED’s application in regular traffic management presents an intriguing avenue for future exploration.

### 5.2. Concluding Remarks

The proposed RTAIAED system is cost-effective and shows good performance and accuracy. Demonstrating substantial practical applicability, it emerges as a versatile solution with the capability to address a multitude of real-world challenges, in particular where other solutions are too complex and expensive to be applied, like managing temporary situations (e.g., road restrictions due to roadworks or because the road is partially blocked by landslides). The envisioned future enhancements not only aim to broaden the system’s scope and integration with other smart technologies but also highlight the potential for significant contributions to emergency response optimization and traffic management in the ongoing evolution of intelligent transportation systems, poised to make meaningful impacts in enhancing safety and efficiency across urban and sub-urban environments.

## Figures and Tables

**Table 1:** Examples of recommended distances for the Lookout Station from the nearest traffic light.

Yellow Time (s)	Vehicle Speed (km/h)	Road Length (m)	Required Time (s)	Optimal Distance (m)
3	50	200	17.4	435
5	70	200	15.2	382
3	50	400	31.8	795
5	70	400	25.6	639
3	50	600	46.2	1155
5	70	600	35.9	896

**Table 2:** Examples of minimum time of green light.

Yellow Time (s)	Vehicle Speed (km/h)	Road Length (m)	Min Green Time 1 (s)	Min Green Time 2 (s)	Optimal Distance (m)
3	50	200	39.2	21.8	435
5	70	200	34.4	19.2	382
3	50	400	71.6	39.8	795
5	70	400	57.5	31.9	639
3	50	600	104.0	57.8	1155
5	70	600	80.6	44.7	896

**Table 3:** Results on the evaluation dataset of the object detector model 480t.

Class	Instances	Precision	Recall	mAP50	mAP50-95
All	1758	81.6%	70.9%	78.1%	54.6%
Ambulance	314	82.9%	89.7%	93.3%	80.0%
Flashing Light	196	76.3%	69.9%	77.0%	43.7%
Star of Life	482	87.7%	71.0%	80.6%	52.5%
Written Ambulanza	130	72.6%	53.8%	58.1%	38.5%
Written Ambulanza Reversed	200	81.1%	82.0%	86.1%	55.9%
Rear Ambulance	161	81.3%	77.0%	86.5%	68.8%
Red Cross	275	89.5%	52.5%	65.3%	42.6%

**Table 4:** Aggregated results on the evaluation dataset of the models 640n, 640t, 480n, 480t.

Model	Precision	Recall	mAP50	mAP50-95
640n	85.7%	76.0%	82.6%	60.6%
640t	88.4%	73.4%	83.5%	59.7%
480n	82.3%	70.0%	77.0%	54.6%
480t	81.6%	70.9%	78.1%	54.6%

**Table 5:** False positives and false negatives of all the models with 0.8, 0.85, and 0.9 thresholds.

Model	FPO-TPA-FPR 0.8	FPO-TPA-FPR 0.85	FPO-TPA-FPR 0.9
640n	3.8%-79.8%-1.9%	3.0%-76.0%-1.6%	2.8%-74.8%-1.6%
640t	3.8%-78.2%-0.9%	3.2%-74.8%-0.9%	3.0%-73.5%-0.9%
480n	3.2%-77.1%-1.6%	2.2%-72.5%-1.6%	2.0%-71.7%-1.3%
480t	2.8%-76.4%-2.2%	2.4%-72.5%-2.2%	2.0%-71.0%-2.2%

**Table 6:** True Positives, False Positives, and False Negatives of the RTAIAED and its elementary detectors alone.

Detector	TP	FP	FN
Ambulance Detector with threshold 0.85	94	21	0
Ambulance Detector with threshold 0.90	92	7	2
Siren Detector	89	11	5
**RTAIAED**	**94**	**0**	**0**

**Table 7:** Comparison of the fps reached by the various models.

Model	Format	FPS Jetson Nano	FPS Raspberry Pi 5
640n	default	6.3	2.0
640n	onnx	6.8	3.9
640t	default	6.9	2.0
640t	onnx	7.8	4.5
480n	default	10.1	2.6
480n	onnx	9.7	7.0
**480t**	**default**	**11.8**	2.8
**480t**	**onnx**	10.1	**8.2**

## Data Availability

Dataset available on request from the authors.

## Abbreviations

The following abbreviations are used in this manuscript:

A.I.	Artificial Intelligence
AIAE	Ambulance in an Emergency
AP	Average Precision
API	Application Programming Interface
FPO	Other vehicle or other parts in an image classified as ambulance
FPr	False Positive rate
FPR	Rear ambulance classified as ambulance
GSM	Global System for Mobile Communications
IoU	Intersection over Union
LS	Lookout Station
NN	Neural Network
MFCCs	Mel Frequency Cepstral Coefficients
PDA	Personal Digital Assistant
PM	Part-Based Models
RFID	Radio Frequency Identification
RTAIAED	Real-Time Ambulance in an Emergency Detector
SUEM	Urgency and Medical Emergency Service
TL	Traffic Light
TLA	Traffic Light A
TLB	Traffic Light B
TLC	Traffic Light Controller
TPA	True Positive of ambulance
TPr	True Positive rate
